# Spatial Distribution, Risk Assessment, and Source Apportionment of Heavy Metals in Soils from the Sorghum Cultivation Base in the Chishui River Basin, China

**DOI:** 10.3390/toxics14060532

**Published:** 2026-06-20

**Authors:** Ziping Pan, Xiu Li, Yilu Yuan, Junchen Zhang, Yuting Jiang, Zengping Ning

**Affiliations:** 1 School of Resources and Environment, Moutai Institute, Renhuai 564500, China; zipingpan@163.com (Z.P.);; 2 Laboratory of Karst Environmental Evolution and Ecological Security, Institute of Geochemistry, Chinese Academy of Sciences, Guiyang 550081, China

**Keywords:** soil heavy metals, ecological risk assessment, source apportionment, sorghum cultivation base for Baijiu spirit, Chishui River Basin

## Abstract

The Chishui River Basin, a core production area for Chinese sauce-aroma Baijiu (exemplified by Moutai), supports sorghum cultivation critical to the liquor’s distinctive quality. The soil environment quality within this region, therefore, directly impacts the safety and quality of both raw material and the final distilled spirit. To underpin the safe production and sustainable development of this iconic beverage, it is essential to assess soil heavy metal contamination in the soils and quantify the contributions from various sources. In this study, 172 surface soil samples were collected from typical sorghum planting bases in the Renhuai area. Concentrations of eight heavy metals (loids) (As, Cd, Cr, Cu, Hg, Ni, Pb, and Zn) were determined. The contamination status was evaluated using the geostatistical inverse distance weighting interpolation, the Nemerow pollution index (*P_N_*), and the potential ecological risk index (*RI*). Source identification and quantification were performed using the positive matrix factorization receptor model (PMF). Results revealed significant enrichment of Cd and Hg in the soil, with mean concentrations 2.07 times and 2.54 times the soil background values for Guizhou Province, respectively. Pollution index results (*P_i_*, *P_N_*) indicated that soil Cd contamination is relatively severe, whereas contamination from other elements is minimal. Overall, approximately 86.5% of the study area was classified as clean or only slightly polluted. Cd poses a moderate ecological risk and was the primary contributor to the total ecological hazard. Other elements exhibited lower risk, resulting in a slight overall potential ecological risk. The soil environmental quality in certified organic sorghum bases was generally favorable. PMF analysis identified three principal sources: historic industrial emissions and traffic-related sources (contributing 46%), weathering of carbonate rocks combined with agricultural activities (37%), and natural background coupled with organic fertilizer application (17%). In conclusion, while the overall soil heavy metal pollution level in the sorghum planting areas is low, the notable enrichment and higher ecological risk of Cd necessitate enhanced dynamic monitoring and targeted risk control measures to ensure long-term soil health and product safety.

## 1. Introduction

Soil is an important natural resource and environmental element for human survival and socioeconomic development, and plays an indispensable role in maintaining global environmental equilibrium and ensuring agricultural safety [1]. However, with the rapid development of industry, agriculture, and urbanization, the heavy metal pollution in soil has become a focal environmental issue of global concern [2,3,4]. Investigating and researching the geochemical characteristics, eco-environmental risks, and major sources of heavy metals in soil not only reveals the influence of various factors on these metals but also holds great significance for managing soil heavy metal pollution and controlling ecological risks [5,6,7].

The overall soil environment in China is critically stressed. According to the Report on the National Soil Contamination Survey (2014), the total exceedance rate of soil pollutants in China was 16.1%, with heavy metals alone accounting for 82.8% of the exceedances [8]. Heavy metal concentrations in agricultural soils generally exceed regional background values, cadmium (Cd) and mercury (Hg) pollution are particularly prominent, and the associated ecological risks cannot be overlooked [9]. Heavy metal pollution in China’s soils has significant spatiotemporal heterogeneity, with cadmium (Cd), mercury (Hg), arsenic (As), and lead (Pb) displaying a gradual increasing trend from the northwest to the southeast and from the northeast to the southwest [10].

With respect to pollution sources, heavy metals in soils can be classified into two major categories, namely anthropogenic inputs and geological origins, and their mobilities differ considerably [11,12,13,14,15,16,17]. In China’s Yangtze River Delta and Pearl River Delta regions, heavy metals are mainly derived from anthropogenic activities, leading to high mobility and ecological risks. In contrast, the karst region of Southwest China, owing to its distinctive geological background, exhibits generally elevated background concentrations of Cd, As, and Pb in soils, thus representing a typical high-geological-background area [18].

Therefore, identifying contamination sources and developing targeted control measures are prerequisites for implementing precise, zonal, and classified soil environmental management. Common methodologies for heavy metals apportionment include correlation analysis [19], principal component analysis (PCA) [20], positive matrix factorization (PMF) model [21,22], and absolute principal component score-multiple linear regression (APCS-MLR) [23,24]. The PMF model, which employs a least squares approach for iterative data analysis, can resolve pollution sources and quantify their contributions without requiring prior source profiles. Owing to this advantage, it has been extensively employed in environmental geochemistry studies in recent years [21,22].

Sorghum (*Sorghum bicolor* orghum bicolor (L.) Moench) is the fifth most important cereal crop globally after wheat, corn, rice, and barley [25]. It serves as an essential raw material for China’s traditional brewing industry, primarily used for producing fermented products such as Baijiu and vinegar [26,27]. ‘Hongyingzi’ sorghum, also recognized as organic sorghum, is an irreplaceable raw material for brewing Moutai liquor. Its cultivation is geographically restricted to Renhuai in Guizhou Province and adjacent regions along the Chishui River Basin, including Jinsha, Xishui, and Bozhou counties. Renhuai City is not only the core production area for Chinese sauce-aroma Baijiu but also the primary cultivation zone for organic sorghum, hosting approximately 300,000 mu (approximately 300 of sorghum planting bases. These bases are regarded as the “first production workshop” for Moutai liquor. Consequently, the concentration and quality of heavy metals in their soils directly influence the safety and quality of sauce-aroma-type Baijiu. Nevertheless, research on the soil environmental quality of these sorghum cultivation bases remains limited. Existing studies have predominantly focused on aspects like soil fertility and nutrient status assessments [28,29], while a systematic analysis of the patterns and sources of heavy metal contamination in soils dedicated to sauce-aroma Baijiu production has yet to be reported.

In this study, the sorghum planting bases in Renhuai City dedicated to Moutai liquor production were selected as the research area. Based on systematic field sampling and elemental analysis, the geochemical features and spatial distribution of heavy metals were analyzed to assess soil environmental quality and potential ecological risks. Multivariate statistical analysis and the PMF receptor model were utilized to identify and quantify the contributions of heavy metal sources. This study aims to offer a scientific basis and data support for managing heavy metal-related environmental risk in the sauce-aroma Baijiu sorghum planting base. The findings are expected to contribute to ensuring the safe supply of brewing raw materials and promoting the sustainable development of the Baijiu industry in Guizhou Province, Southwest China.

## 2. Study Area

**Figure 1:**
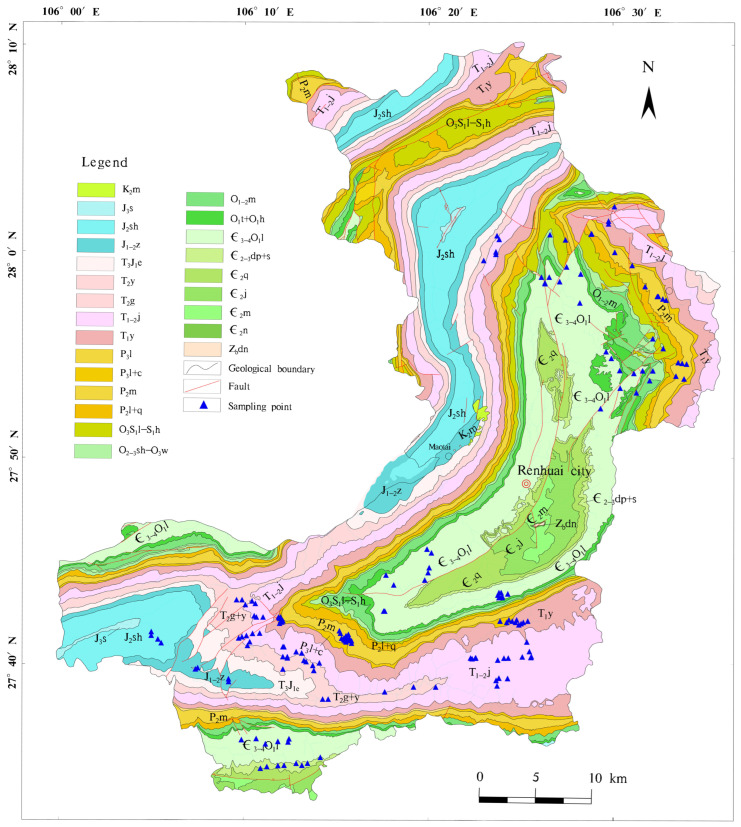
Distribution map of major strata and sampling sites in the study area.

Renhuai City (27° N, 106° E), located in the western part of Zunyi City, Guizhou Province, Southwest China, covers a total area of approximately 1788 km^2^ and is renowned as the birthplace of Maotai—a globally acclaimed Chinese baijiu. The terrain descends stepwise from southeast to northwest, with altitudes ranging from 1000 to 1600 m above sea level. The region experiences a mid-subtropical humid monsoon climate [21]. Geotectonically, the study area lies within the Tongzi-Bijie Early Paleozoic Fold-Thrust Belt of the Upper Yangtze Platform. Exposed strata primarily include Ordovician, Cambrian, Permian, Triassic, and Jurassic systems, with Triassic and Jurassic strata being most widely distributed. The lithosphere is dominated by claystone, siltstone, sandstone, limestone, and dolomite (Figure 1). Predominant soil types are yellow soils and limestone soils [30]. Land use is primarily forest and cultivated land. The local sorghum cultivar “Hongyingzi” (organic sorghum), the core raw material for Moutai liquor, is widely cultivated across approximately 20,000 hectares.

## 3. Materials and Methods

### 3.1. Sampling Process

Based on the actual distribution of organic sorghum cultivation bases, representative areas were selected for soil sample collection. The locations of all sampling sites are presented in Figure 1. Field sampling was conducted in accordance with the Chinese national standard (HJ/T 166-2026) [31]. Within each selected sorghum base, typical plots were designated as sampling units. For each unit, 3–5 soil subsamples were collected from the topsoil layer (0–20 cm depth) within a 50 m diameter area. The subsamples were thoroughly mixed, quartered, and reduced to obtain a composite sample of approximately 1.5 kg representing the unit. Each composite sample was then placed in a labeled canvas bag. The geographic coordinates of each sampling point were recorded in situ using a GPS device. Concurrently, field observations including soil color, type, and local fertilization practices were documented.

All soil samples were air-dried naturally at ambient temperature. Visible stones, debris, and other impurities were manually removed. The dried samples were gently ground using a wooden hammer or roller and passed through a 20-mesh sieve. The sieved soil was then quartered, and approximately 500 g was transferred into pre-cleaned polyethylene bottles, sealed, and stored for further analysis. For heavy metal determination, a 100 g aliquot was finely ground using an agate mortar to pass through a 100-mesh sieve (particle size ≤ 0.149 mm).

### 3.2. Sample Analysis

Eight heavy metals were analyzed per sample: zinc (Zn), lead (Pb), cadmium (Cd), chromium (Cr), nickel (Ni), copper (Cu), and arsenic (As). All the reagents used for the digestion of samples were of superior purity grade.

(1) Determination of Zn, Pb, Cd, Ni, Cu, As, and Cr levels in soils

Precisely 0.2000 g (accurate to 0.0001 g) of the sieved soil sample was weighed into a polytetrafluoroethylene (PTFE) digestion vessel. The sample was first predigested by adding 6 mL of HNO_3_, 1 mL of HF, and 2 mL of HCl, followed by a 20 min standing period. Subsequently, Microwave-assisted digestion was performed using a microwave digestion system (CEM Mars 6, CEM Corporation, Matthews, NC, USA)and the temperature program for Heavy metal digestion (except Hg) was as follows: ramp to 120 °C over 5 min and hold for 7 min; ramp to 150 °C over 5 min and hold for 5 min; ramp to 190 °C over 5 min and hold for 40 min, then cool to below 50 °C. After digestion, the vessel was transferred to an acid fume exhaust apparatus and heated at 140 °C to evaporate the solution to approximately 1 mL. Finally, each sample was filtered into a 50 mL volumetric flask for analysis. The concentrations of the digestate were measured using inductively coupled plasma mass spectrometry (ICP-MS, NexION 1000G, PerkinElmer, Waltham, MA, USA). Under the optimal instrument conditions, the analysis was performed on the KED (kinetic energy discrimination) mode by using He as the collision gas. This method was chosen as it can be applied to almost all interferences using a single set of operating conditions and does not require an in-depth knowledge of the sample prior to analysis.

(2) Determination of Hg concentration and pH in soils

Precisely 0.5000 g (accurate to 0.0001g) of the prepared soil sample was placed into a digestion vessel. A mixture of 2 mL HNO_3_ and 6 mL HCl was added, followed by microwave-assisted digestion at 180 °C and held for 25 min. After cooling, the digest was quantitatively transferred and diluted with ultrapure water to 50 mL. Depending on the expected mercury concentration, the sample solution was further diluted 2- to 10-fold. Mercury content was determined using an atomic fluorescence spectrometer (AFS-921, Beijing Jitian Instruments Co., Ltd.,Bejing, China) in accordance with the Chinese national standard HJ 680-2013.

Soil pH (1:2.5 distilled H_2_O) was measured with a pH meter (Leici PHS-3C, Shanghai INESA Scientific Instrument Co., Ltd., Shanghai, China) [32].

(3) Quality control and assurance

The concentrations of heavy metals were determined using standard national analytical methods. The method detection limits (MDLs) are listed in Table 1. For quality assurance and control, five replicate samples and two certified reference materials (GSS-4a and GSS-5a) were analyzed per batch of 50 samples. The relative errors of certified reference materials and parallel samples are all less than 10%, which ensures the accuracy and repeatability of the sample analysis results [33].

### 3.3. Nemerow Integrated Pollution Index Analysis

To evaluate the combined pollution level of multiple heavy metals in soil, the Nemerow integrated pollution index (*P_N_*) was calculated using the following equations [34]:

*P_i_* = *C_i_*/*S_i_* (1)

*P_N_* = [(*P_i_*_,*max*_^2^ + *P_i_*_,*ave*_^2^)/2]^1/2^ (2)

where *P_i_* represents the single-factor pollution index of an individual element; *C_i_* is the measured concentration of that element in soil (mg/kg); *S_i_* denotes the corresponding limit value specified in National Soil Environmental Quality Standard (GB 15618-2018) [35]; *P_i_*_,*ave*_ is the average of all *P_i_* values; *P_i_*_,*max*_ is the maximum *P_i_* value. The classification criteria for pollution levels are presented in Table 2.

### 3.4. Ecological Risk Assessment of Heavy Metals

The risk index (*RI*), originally proposed by the Swedish scholar Hakanson, was applied to assess the potential ecological risks posed by heavy metals in the soil [36]. This index integrates the toxicity coefficients of heavy metals, their environmental mobility, and the susceptibility of soil ecosystem. The calculation was performed using the following equation:

(3) $${RI=\sum_{i=1}^{n}E}_{r}^{i}={\sum_{i=1}^{n}T}_{r}^{i}\times c_{m}^{i}/c_{b}^{i}$$

where $E_{r}^{i}$ represents the potential ecological risk index for an individual metal *i*; $c_{m}^{i}$ is the measured concentration of metal *i* in the soil (mg/kg); $c_{b}^{i}$ is the corresponding regional soil background value [37]; $T_{r}^{i}$ is its toxicity response coefficient (Cr = 2, As = 10, Cu = 5, Cd = 30, Zn = 1, Pb = 5, Ni = 5, Hg = 40). The classification criteria for ecological risk levels are presented in Table 3.

### 3.5. The Positive Matrix Factorization (PMF) Receptor Model

PMF 5.0 receptor model, developed by the U.S. Environmental Protection Agency (US EPA), was employed for source apportionment. As a widely used multivariate analysis tool, it decomposes the measured elemental concentration matrix (*X*) into three component matrices: a source profile matrix (*F*) representing the chemical composition of each pollution source, a source contribution matrix (*G*) indicating the contribution of each source to every sample, and a residual matrix (*E*) containing unexplained variation [38]. The basic model structure is expressed as:

(4) $$X_{ij}=\sum_{i=1}^{p}(G_{ik}\times F_{ik})+E_{ij}$$

where *p* represents the number of pollution sources. The model iteratively determines the optimal solution by minimizing the objective function *Q*.

(5) $$Q=\sum_{i=1}^{n}\;\sum_{j=1}^{m}{\;\left(\frac{E_{ij}}{U_{ij}}\right)}^{2}$$

The uncertainty *U_ij_* is calculated as follows:

(6) $$U_{ij}=\left\{\begin{array}{cc} (5/6)MDL, & \;\;\;c\leq MDL\\ \sqrt{{\left(\delta \times c\right)}^{2}+{\left(0.5\times MDL\right)}^{2}}, & \;\;\;c>MDL \end{array}\right.$$

where *m* is the number of elements analyzed; *n* is the total number of samples; *Q* is the objective function to be minimized; *U_ij_* is the estimated uncertainty for element *j* in sample *i*; *δ_ij_* is the relative standard deviation associated with the measurement; and *C_ij_* is the measured concentration of element j in sample *i* (mg/kg).

The method detection limits (MDLs) are listed in Table 1.

### 3.6. Statistical Analysis and Geochemical Mapping

Microsoft Excel 2016, Origin 2024, and SPSS 26.0 were used for descriptive statistical analysis and correlation analysis. EPA PMF 5.0 was used for source apportionment of heavy metal pollution in soil.

MapGIS 6.7 was utilized for geological mapping of sampling sites (Figure 1). Maps that show spatial distributions of heavy metals and the Nemerow pollution index were generated by using ArcGIS 10.8 (Environmental Systems Research Institute, Redlands, CA, USA).

## 4. Results and Discussion

### 4.1. Spatial Distribution Characteristics of Heavy Metals in Surface Soil

The concentrations of elements in the soil samples are summarized in Table 4. The mean concentrations of Cr, Cd, As, Cu, Hg, Ni, Pb, and Zn were 100.22, 0.53, 16.34, 44.57, 0.17, 40.39, 32.44, and 88.59 mg/kg, respectively, all of which exceeded the corresponding national and Guizhou provincial background values for surface soil [39]. The enrichment factors, i.e., K values (ratio to Guizhou surface soil background value), for As, Cd, Zn, Cr, Cu, Hg, Ni, and Pb were 1.02, 2.54, 1.02, 1.19, 1.66, 2.07, 1.23, and 1.04, respectively. Notably, Hg and Cd exhibited the most significant enrichment, with mean concentrations approximately twice the Guizhou background, indicating pronounced anomalous distribution. The coefficients of variation (CV) ranged from 0.32 to 0.89. The CV values for Cr, Cd, As, and Cu exceeded 0.5, suggesting high spatial heterogeneity that may be attributed to varying degrees of exogenous inputs. According to the Chinese Soil Environmental Quality Standard [35], the average Cd content (0.53 mg/kg) exceeded the risk screening value (0.3 mg/kg, pH ≤ 7.5). At one sampling point, Cd content also exceeded the corresponding risk intervention value. Concentrations of the other seven elements remained below their respective risk screening values at all sampling points, with no exceedance of intervention thresholds. Nevertheless, given the considerable spatial variability of elements such as As and Cd, localized pollution risks cannot be ruled out. Heavy metal concentrations in soils of the study area exceed the background values of both Guizhou Province and China as a whole. They are comparable to those in adjacent Xishui County [40], significantly lower than those in Heng County (Guangxi) [41] and northeast Yunnan [42], but much higher than those in the black soil region of Yushu, Jilin Province [43]. This phenomenon is primarily attributed to the high natural background of heavy metals in carbonate rocks and the secondary enrichment that occurs during weathering processes. Based on the main outcropping strata in the region, the high-value areas of Cd and Hg are mainly concentrated in the limestone distribution zones of the Permian Qixia Formation–Maokou Formation and the Triassic Yelang Formation–Guanling Formation in the northeastern part of the study area. This spatial pattern is consistent with previous findings [44]. The origin of this enrichment is closely associated with the soil formation process from carbonate rock weathering: carbonate minerals are rapidly dissolved and substantially leached, leading to a marked reduction in the volume of weathering products, whereas most heavy metal elements remain in the residues. Consequently, heavy metals tend to be relatively enriched in soils over carbonate rock regions [45]. Furthermore, the inappropriate application of chemical fertilizers during early agricultural practices, such as sorghum cultivation, also contributes to cadmium accumulation in the soil.

**Figure 2:**
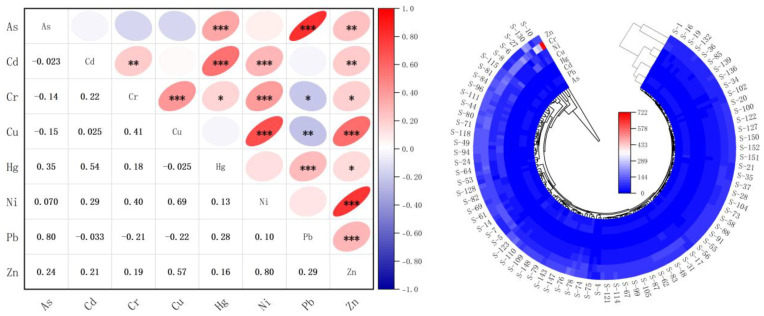
Correlation heatmap (**left**) and hierarchical clustering dendrogram (**right**) of heavy metals. Note: * represents *p* < 0.05, ** represents *p* < 0.01, *** represents *p* < 0.001.

Correlation analysis is commonly used to identify potential common sources among different elements [46]. Hierarchical cluster analysis provides a more intuitive visual representation of elemental groupings based on their similarities [47]. As shown in the correlation matrix (Figure 2, left), significant positive correlations (*p* < 0.01, correlation coefficient > 0.60) were observed between As and Pb, Ni and Zn, and Cu and Ni, suggesting these pairs likely share similar origins or exhibit comparable geochemical behavior. Several other element pairs (As–Hg, Cd–Hg, Cr–Cu, Cd–Ni, Cr–Ni, Cu–Zn, Hg–Pb, and Pb–Zn) also showed statistically significant positive relationships (*p* < 0.05). The results of hierarchical cluster analysis (Figure 2, right) indicated that the eight heavy metals could be classified into four distinct clusters. Cr and Zn each formed separate clusters, whereas Cu and Ni were grouped together, supporting their strong geochemical association inferred from correlation analysis. The clustering of As, Hg, Pb, and Cd into one group implies that these elements may originate from more complex or mixed sources, possibly reflecting composite pollution influences.

### 4.2. Spatial Variations in the Elemental Levels

**Figure 3:**
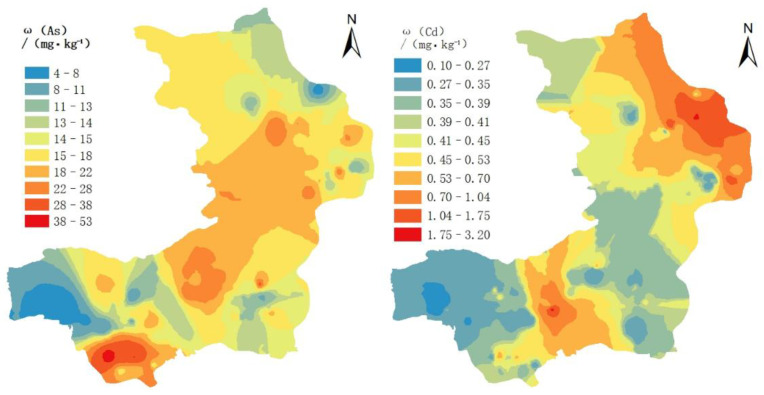

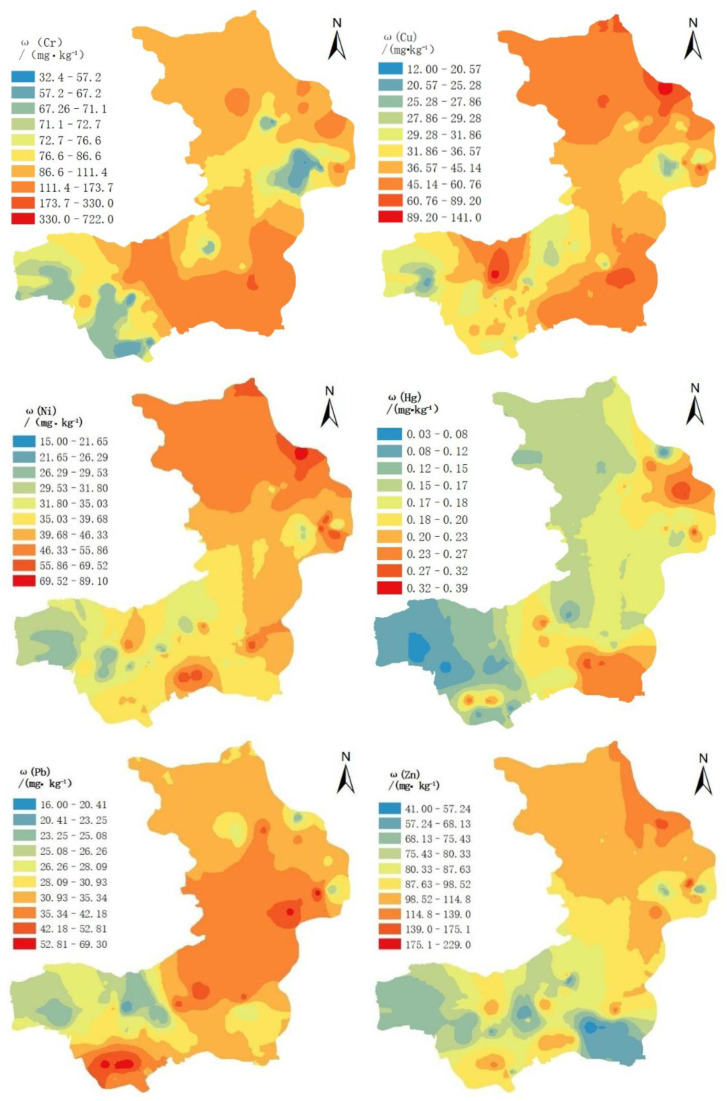
Predicted spatial distribution maps of elements in the soils across the study area.

Figure 3 presents the spatial distribution patterns of the studied heavy metals in the soils of the sorghum cultivation bases. Pb and As exhibited similar spatial trends, with higher concentrations predominantly observed in the southern Houshan Township and along a central belt extending through Luban, Zhongshu, Canglong, and Gaodaping. Conversely, lower concentrations of these elements were mainly clustered in the southwestern Jiucang-Longjing area and the northeastern Huoshigang region.

The spatial distribution of cadmium Cd showed elevated concentrations primarily in the southern Wuma-Luban area and the northeastern Xuekong-Huoshigang zone. This spatial distribution pattern exhibits a high degree of spatial correlation with the outcrop area of the Permian Maokou Formation, indicating a strong association between elevated Cd levels and this specific geological setting.

Cr, Zn, Cu, and Ni contents generally shared a similar spatial variation trend. Their high-concentration zones were largely concentrated in southern Renhuai, encompassing the Tanchang-Changgang-Wuma-western Luban area, as well as the junction of Xuekong, Daba, and Erhe towns. The distribution of Cu was relatively more extensive, with medium to high concentration areas also present in the northern part of the study area.

The high-concentration areas of Hg were relatively limited and dispersed, mainly appearing in Changgang and most parts of Xuekong in the south, as well as in the localized patches within Gaoping, Xitou, Luban, and Houshan.

### 4.3. Pollution Characteristics and Ecological Risks of Heavy Metals

#### 4.3.1. Nemerow Integrated Pollution Indices

**Figure 4:**
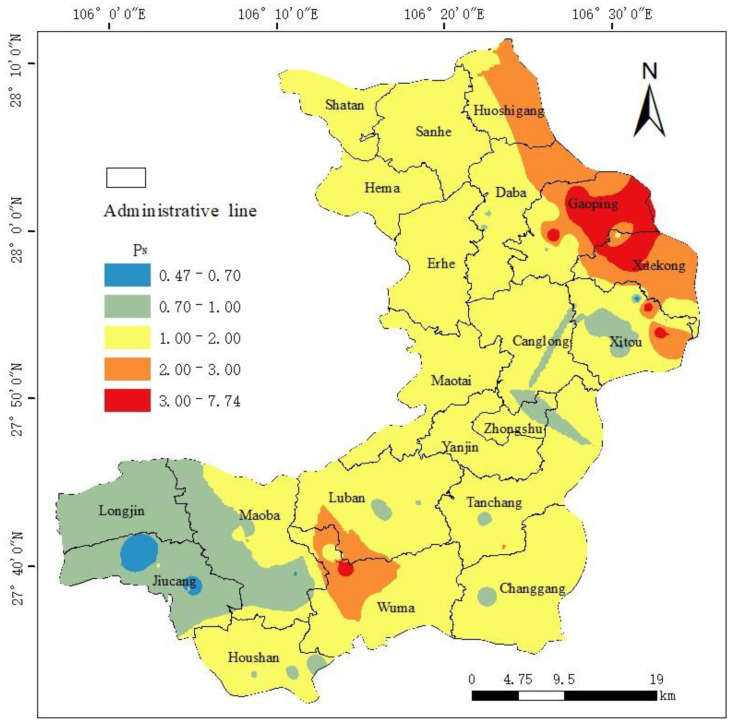
Spatial distribution map of the Nemerow pollution index for the heavy metals in the research site.

The evaluation results based on *P_N_* and *P_i_* values are summarized in Figure 4 and Table 5. Among all studied heavy metals, Cd was identified as the primary pollutant. The *P_i_* values for Cd (*P_Cd_*) were 0.43–10.78, with a mean of 1.78, demonstrating a slight overall contamination level. Over 75% of the sampling sites exhibited Cd contamination (*P_Cd_* > 1), and approximately 11.04% of the sites were classified as severely contaminated (*P_Cd_* > 3). These findings indicate that Cd pollution is a significant local concern.

In contrast, the average *P_i_* values for the other seven elements were all below 1.0, and over 70% of sampling sites were classified as clean for these elements. However, localized slight to moderate pollution was still observed for As, Cr, Cu, and Ni.

The *P_N_* values across the study area varied from 0.47 to 7.74 (mean: 1.44), reflecting an overall slight composite pollution status. Approximately 40% of the sampling sites were classified as clean (*P_N_* ≤ 0.7), while nearly 60% exhibited some degree of pollution. Notably, about 13.53% of the area was characterized by moderate or severe pollution levels. Spatial comparison revealed a strong consistency between the distribution pattern of *P_N_* and the geochemical anomalies of Cd, confirming that the comprehensive soil pollution in the study area is primarily driven and spatially controlled by elevated Cd concentrations.

#### 4.3.2. Ecological Risks of Heavy Metals in Soil

Based on the risk screening values specified in the “Soil Pollution Risk Control Standard for Agricultural Land”, the studied heavy metals were calculated (Table 6). The mean *E_r_* values were, in descending order: Cd (53.40) > Ni (13.89) > As (8.17) > Pb (3.37) > Cr (3.34) > Zn (2.32) > Cu (2.23) > Hg (0.44). Cd presented the highest potential risk, with *E_r_* values ranging from 13.00 to 323.5, corresponding to a moderate risk level. Approximately 52.60% of the sampling sites were classified as low risk, 34.42% sites as moderate risk, and 12.98% as high, severe, or very severe risk levels.

For the other seven heavy metals, the average and maximum *E_r_* values were all below 40, indicating a low ecological risk level. The RI values for all heavy metals ranged from 26.50 to 361.0, with a mean value of 81.60. Over 90% of the sampling sites had RI values below 150, classifying them as low ecological risk areas, while the remaining sites (approximately 8.82%) exhibited a medium risk status. Overall, the cumulative potential ecological risk posed by heavy metals in the study area is low, and the soil environment quality of the organic sorghum cultivation base is generally good. Heavy metal-related environmental concerns were not significant at most sampling locations.

### 4.4. Source Apportionment

**Figure 5:**
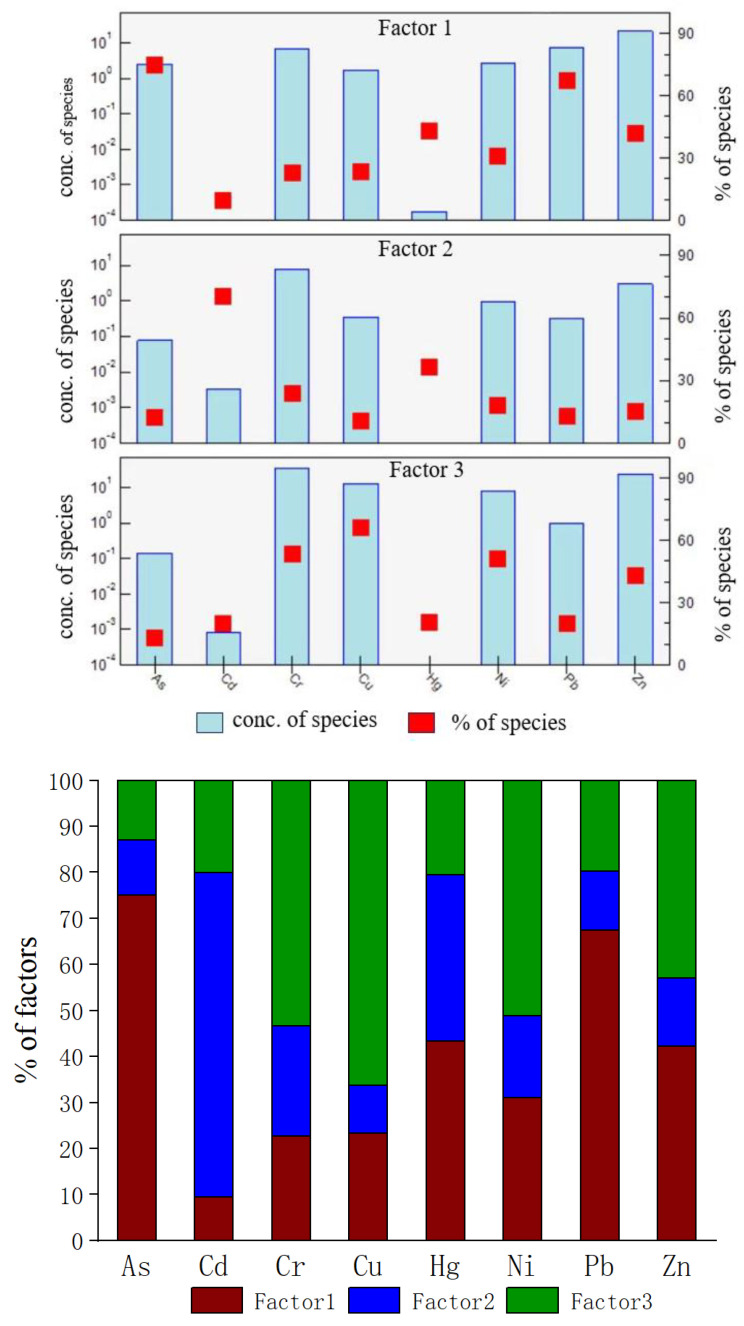
Contributions of different heavy metal sources in sorghum base soils in the study area.

The source apportionment of heavy metals was performed using the EPA PMF 5.0 model by inputting concentration data and associated uncertainties. The model was run with 20 random initial starting points to avoid local minima, and the optimal solution was selected based on the smallest Q value. Comparing the Q values and examining the scaled residuals for factor numbers ranging from 2 to 6, a three-factor solution was identified as optimal. The coefficient of determination (R^2^) for individual elements ranged from 0.59 to 1.00, with the robust Q (Q_robust_) approximating the theoretical Q (Q_true_). Most residuals fell within the range of −3 to 3, indicating that the three-factor model adequately explained the observed data [11,22,24]. The source profiles and contributions are illustrated in Figure 5.

Factor 1 was characterized by high loadings of Pb (67.52%), As (75.08%), and Hg (43.39%), while contributions of other heavy metals remained below 42.3%. Arsenic is a well-documented tracer for industrial emissions, often associated with wastewater and solid waste from metallurgical, chemical, and mining operations. Lead is a typical marker for traffic-related sources, derived from vehicle exhaust, tire wear, and mechanical abrasion. Its elevated contribution likely reflects historical deposition from leaded gasoline or long-term accumulation near major traffic arteries. The moderate contribution of Hg may be attributed to atmospheric deposition from industrial coal combustion (e.g., power plants and industrial boilers). Therefore, Factor 1 was interpreted as historical industrial and traffic emissions, accounting for 36.8% of the total contribution.

Factor 2 was dominated by Cd, which contributed 70.35%, whereas the contributions of the other seven elements were all below 36.2%. The study area is situated in a karst region where intense weathering of carbonate rocks leads to significant leaching of alkaline earth elements such as Ca and Mg [48]. The resulting neutral to alkaline soil conditions (pH > 6) promote the specific adsorption and retention of Cd by clay minerals and organic matter, leading to its progressive enrichment [11]. In addition, Cd is a common impurity in phosphate fertilizers, and long-term excessive application may lead to its accumulation in soils. The use of agricultural plastics and certain pesticides (e.g., some fungicides) can also introduce Cd into the soil system [49]. Thus, Factor 2 was identified as carbonate rock weathering/pedogenic processes coupled with agricultural activities (fertilization and pesticides), contributing approximately 17.1%.

Factor 3 showed prominent contributions from Cu (66.18%), Cr (53.30%), Ni (51.02%), and Zn (42.87%). These elements are commonly abundant in soil parent materials [12] and generally exhibit high natural background values in the region. Their contributions exceeding 50% largely reflect the inherent geochemical characteristics of the local soil. The notably high contribution of Cu (66.18%) is also closely linked to the application of organic fertilizer such as livestock manure, which has been recognized as a significant pathway for Cu and Zn input in agricultural systems [50]. Consequently, Factor 3 was attributed to natural geological background combined with organic fertilizer application, accounting for 46.1% of the total contribution.

In summary, the PMF analysis revealed that heavy metals in the soils of the Moutai liquor organic sorghum base primarily originated from three sources: historical industrial and traffic sources (36.8%), carbonate rock weathering and agricultural inputs (17.1%), and natural background enhanced by organic fertilizer application (46.1%). Particular attention should be given to the first and third source categories. Implementing scientifically sound control measures is essential to mitigate potential ecological risks. However, as this study did not measure heavy metal concentrations in sorghum plants or Baijiu products, the direct transfer of these heavy metals to the final beverage remains to be quantified in future research.

## 5. Conclusions

This study systematically assessed the contamination status, ecological risks, and sources of heavy metals in soils within the organic sorghum cultivation base dedicated to Moutai liquor production. The main conclusions are as follows:

(1) Significant enrichment of cadmium (Cd) and mercury (Hg) was observed in the soils of the study area. Their average concentrations were 2.54 and 2.07 times the soil background values of Guizhou Province, respectively. With the exception of Cd, the concentrations of all other elements remained below the risk screening values specified in the Soil Environmental Quality Risk Control Standard for Agricultural Land (GB 15618-2018). However, localized slight to moderate contamination was identified for Cd, As, Cu, and Cr.
(2) Cd was identified as the primary pollutant in the study area. Approximately 60% of the area exhibited a slight or higher level of pollution, with about 13% reaching moderate or severe levels. Spatially, the overall soil pollution pattern was predominantly controlled by the distribution of Cd.
(3) Cd was the only element presenting a moderate potential ecological risk and served as the main contributor to regional ecological risk. Nevertheless, the comprehensive potential ecological risk posed by all studied heavy metals was low. This indicates that the current soil environmental quality of the organic sorghum base is generally good and that the associated ecological risks are largely manageable.
(4) The sources of heavy metals were apportioned to three main categories: historical industrial and traffic emissions (contributing 46.1%), carbonate rock weathering/pedogenesis processes combined with agricultural activities (17.1%), and natural background coupled with inputs from agricultural organic fertilizers (36.8%). To minimize potential risks to brewing raw materials, subsequent environmental management should focus on strengthening source control of key elements such as Cd, Hg, and As. However, direct measurements of heavy metals in sorghum grains and Baijiu products are needed to establish the actual transfer pathways and risks.

## Figures and Tables

**Table 1:** MDLs of elements (mg/kg).

Element	Hg	As	Cd	Cr	Cu	Ni	Pb	Zn
MDL	0.002	0.2	0.03	2	0.7	2	1	5

**Table 2:** Grading criteria for the single-factor and Nemerow integrated pollution indices.

Grade	*P_i_*	Pollution Level	*P_N_*	Pollution Level
1	≤v	Clean	≤0.7	Clean
2	1–2	Slight	0.7–1.0	Relatively Clean (Warning)
3	2–3	Moderate	1.0–2.0	Slight
4	>3	Severe	2.0–3.0	Moderate
5			>3.0	Severe

**Table 3:** Classification criteria for ecological risk levels of heavy metals.

Risk Grade	$E_{r}^{i}$	Risk Level	*RI*	Risk Level
1	<40	Low	<150	Low
2	40–80	Moderate	150–300	Moderate
3	80–160	Considerable	300–600	Considerable
4	160–320	High	>600	High
5	>320	Very High		

**Table 4:** Descriptive statistical analysis of the elemental contents in the samples.

Parameter	As	Cd	Cr	Cu	Hg	Ni	Pb	Zn	pH
Min	4.20	0.13	32.40	12.00	0.03	15.00	16.00	41.00	4.60
Max	53.10	3.24	722.00	141.00	0.39	89.10	69.30	229.00	8.28
Mean	16.34	0.53	100.22	44.57	0.17	40.39	32.44	88.59	6.98
Std. Deviation	8.56	0.48	65.68	24.68	0.07	14.70	11.33	28.34	0.74
CV	0.52	0.89	0.66	0.55	0.40	0.36	0.35	0.32	0.11
Xishui county, Guizhou province [40]	10.80	0.47	88.70	40.00	0.13	43.40	33.10	97.9	8.94
Heng xian, Guangxi province [41]	75.8	1.91	467.0	48.5	0.21	76.2	84.2	258.2	/
Lishu county, Jilin province [43]									
Country background values [39]	11.20	0.097	66.00	22.60	0.065	26.90	26.00	74.20	/
Guizhou background values [39]	16.00	0.21	84.40	26.90	0.084	32.90	31.30	86.90	/
K	1.02	2.54	1.19	1.66	2.07	1.23	1.04	1.02	/
Risk screening value [39]	30	0.3	200	100	2.4	100	120	250	6.5~7.5
	25	0.6	250	100	3.4	190	170	300	>7.5
Risk control value [39]	120	3.0	1000	/	4.0	/	700	/	6.5~7.5
	100	4.0	1300	/	6.0	/	1000	/	>7.5

Note: Heavy metal content is expressed as mg/kg, and pH is dimensionless.

**Table 5:** Nemerow integrated pollution indices for soil heavy metals.

Parameter	Pollution Index			Percent of Pollution Level (%)			
	Range	Mean	Pollution Level	<1.0	1.0~2.0	2.0~3.0	≥3.0
				Clean	Slight Pollution	Moderate Pollution	Severe Pollution
*P_As_*	0.21~2.66	0.82	Clean	79.41	18.24	2.35	0.00
*P_Cd_*	0.43~10.78	1.78	Slight Pollution	23.38	57.79	7.79	11.04
*P_Cr_*	0.22~4.81	0.67	Clean	89.61	9.09	0.65	0.65
*P_Cu_*	0.24~2.82	0.89	Clean	74.12	21.76	4.12	0.00
*P_Hg_*	0.06~0.78	0.35	Clean	100.00			
*P_Ni_*	0.25~1.49	0.67	Clean	86.47	13.53	0.00	0.00
*P_Pb_*	0.23~0.99	0.46	Clean	100.00			
*P_Zn_*	0.21~1.15	0.44	Clean	99.41	0.59	0.00	0.00
*P_N_*	0.47~7.74	1.44	Slight Pollution	40.59	45.88	4.12	9.41

**Table 6:** Potential ecological risks of heavy metals.

Parameter	*E_r_/RI*			Percent of Risk Level (%)				
	Range	Mean	Risk Level	Low	Moderate	Considerable	High	Very High
*E_r_*_(As)_	2.10~26.55	8.17	Low	100	0.00	0.00	0.00	0.00
*E_r_*_(Cd)_	13.00~323.5	53.40	Moderate	52.60	34.42	7.14	5.19	0.65
*E_r_*_(Cr)_	1.08~24.07	3.34	Low	100	0.00	0.00	0.00	0.00
*E_r_*_(Cu)_	0.60~7.05	2.23	Low	100	0.00	0.00	0.00	0.00
*E_r_*_(Hg)_	0.21~1.15	0.44	Low	100	0.00	0.00	0.00	0.00
*E_r_*_(Ni)_	2.40~31.2	13.89	Low	100	0.00	0.00	0.00	0.00
*E_r_*_(Pb)_	1.25~7.43	3.37	Low	100	0.00	0.00	0.00	0.00
*E_r_*_(Zn)_	1.14~4.95	2.32	Low	100	0.00	0.00	0.00	0.00
*RI*	26.50~361.0	81.60	Low	90.59	8.82	0.59	0.00	/

## Data Availability

The original contributions presented in this study are included in the article. Further inquiries can be directed to the corresponding authors.
